# Rapid neural coding in the mouse retina with the first wave of spikes

**DOI:** 10.1186/1471-2202-15-S1-P120

**Published:** 2014-07-21

**Authors:** Geoffrey Portelli, John Barrett, Evelyne Sernagor, Timothée Masquelier, Pierre Kornprobst

**Affiliations:** 1 Neuromathcomp, INRIA, Sophia Antipolis, 06902, France; 2 Institute of Neuroscience, Medical School, Newcastle University, Newcastle UK; 3 Institut de la Vision, UPMC Université Paris 06, Paris, 75012, France; 4 CNRS, UMR 7210, Paris, 75012, France

## 

For flashed stimuli presentations, it is known that the latency of the first spike of retinal ganglion cells (RGC) encodes information about the stimulus. This was shown at the level of individual neurons but also at the population level by considering the relative latency between certain pairs of RGC [1]. In this work, we further investigated this population code on mouse retinas using a 60-channel MEA (469 RGC pooled from 5 retinas) in response to gratings of varying phase, spatial frequency.

Interestingly, due to the presence of a high spontaneous activity, we did not find any RGC pair showing a clear relation between the relative latencies and stimuli as in [1]. So we extended this analysis to the whole population instead of looking at individual pairs, by considering the relative order of all spike latencies, i.e. the shape of the first wave of spikes (FWS) after stimulus onset.

We first showed that the FWS is specific to each stimuli. To do so, we defined a distance between the FWS of a reference grating and the gratings with similar spatial frequency and varying phases. This distance was correlated to the phase difference between gratings.

Then, to estimate quantitatively the coding efficiency of the FWS, we performed a discrimination task where the aim was to identify the phase among gratings of identical spatial frequency. We compared the performance (fraction of correct predictions, FCP) of the FWS under classical Bayesian decoders to independent response latency of each recorded RGC. Results showed the FWS decoder which is based on the relative rank of latencies only, was as efficient as the pure latency decoder based on absolute latency values (~73% of FCP for both).

Finally, as the spikes from the output of the retina are conveyed and processed by higher neural structure such as the Lateral Geniculate Nucleus (LGN), we investigated the possible effects of an a posteriori processing stage on the neural code. We fed a simulated LGN-like layer [2] with the spikes obtained from our recordings. We then analyzed the output spikes from the simulated LGN using the same discrimination task. As the number of RGC increased, the FWS decoder rapidly outperforms the latency decoder. Considering all RGC, we compared the performance obtained before and after the LGN. Results showed the latency decoder discrimination performance decreased (from 73% to 40% of FCP) although the FWS decoder discrimination performance remained more stable (from 73% to 70% of FCP).
